# An mRNA expression signature for prognostication in *de novo* acute myeloid leukemia patients with normal karyotype

**DOI:** 10.18632/oncotarget.5390

**Published:** 2015-10-23

**Authors:** Ming-Kai Chuang, Yu-Chiao Chiu, Wen-Chien Chou, Hsin-An Hou, Mei-Hsuan Tseng, Yi-Yi Kuo, Yidong Chen, Eric Y. Chuang, Hwei-Fang Tien

**Affiliations:** ^1^ Department of Laboratory Medicine, National Taiwan University Hospital, Taipei, Taiwan; ^2^ Graduate Institute of Biomedical Electronics and Bioinformatics, National Taiwan University, Taipei, Taiwan; ^3^ Department of Internal Medicine, National Taiwan University Hospital, Taipei, Taiwan; ^4^ Bioinformatics and Biostatistics Core, Center of Genomic Medicine, National Taiwan University, Taipei, Taiwan; ^5^ Greehey Children's Cancer Research Institute, University of Texas Health Science Center at San Antonio, San Antonio, Texas, United States of America; ^6^ Department of Epidemiology and Biostatistics, University of Texas Health Science Center at San Antonio, San Antonio, Texas, United States of America

**Keywords:** acute myeloid leukemia, normal cytogenetics, mRNA signature, prognosis

## Abstract

Although clinical features, cytogenetics, and mutations are widely used to predict prognosis in patients with acute myeloid leukemia (AML), further refinement of risk stratification is necessary for optimal treatment, especially in cytogenetically normal (CN) patients. We sought to generate a simple gene expression signature as a predictor of clinical outcome through analyzing the mRNA arrays of 158 *de novo* CN AML patients. We compared the gene expression profiles of patients with poor response to induction chemotherapy with those who responded well. Forty-six genes expressed differentially between the two groups. Among them, expression of 11 genes was significantly associated with overall survival (OS) in univariate Cox regression analysis in 104 patients who received standard intensive chemotherapy. We integrated the z-transformed expression levels of these 11 genes to generate a risk scoring system. Higher risk scores were significantly associated with shorter OS (median 17.0 months vs. not reached, *P* < 0.001) in ours and another 3 validation cohorts. In addition, it was an independent unfavorable prognostic factor by multivariate analysis (HR 1.116, 95% CI 1.035~1.204, *P* = 0.004). In conclusion, we developed a simple mRNA expression signature for prognostication in CN-AML patients. This prognostic biomarker will help refine the treatment strategies for this group of patients.

## INTRODUCTION

“Precision medicine” has become a state-of-the-art principle in clinical care. As a highly heterogeneous disease, acute myeloid leukemia (AML) requires precise risk stratification to achieve optimal treatment outcomes for the patients. Although several clinical and genetic factors have been widely incorporated into clinical consideration for choosing treatment regimens, more prognostic factors would be welcome as there are still factors not yet being sorted out completely. Cytogenetics has long been considered the most important prognostic factor for AML, however, about one-half of the patients are cytogenetically normal (CN); this group of patients need further prognostic factors for risk stratification [1]. Recently, several genetic mutations with prognostic significance, such as internal tandem duplication of *FLT3* (*FLT3*-ITD) [2–4], *NPM1*, and *CEBPA* mutations [5, 6], have partially compensated for the problem. However, about 24% CN-AML patients have no detectable mutations in these genes [2]. Although the expression levels of genes such as *BAALC* [7], *MN1* [8], and *ERG* [9] provide further reference for prognostication in this group of patients, the significance of single gene expression remains restrictive in the context of a complicated cellular milieu.

DNA microarray technology makes it possible to evaluate the global gene expression profiling of cells. Studies have shown distinct genetic expression profiles in AML with different cytogenetics and gene mutations [10–12]. While gene expression signature-derived scoring systems bear prognostic values in AML [11, 13–20], it is rarely used in clinical practice, mainly because of the large gene numbers in those scoring systems, usually dozens to hundreds of probes. For example, Shaughnessy *et al*. developed a 70-gene expression scoring system to identify patients with shorter progression-free survival (PFS) and overall survival (OS) in multiple myeloma [21]. Subsequently they simplified the system to 5 genes which carried the most discriminatory power of the 70-gene risk model with similar predictive values [22].

We realize that a considerable portion of CN-AML patients still need reliable parameters for choosing optimal treatment strategies. In this study, we developed a simple gene expression signature with prognostic significance by incorporating limited number of probes through comprehensive analysis of the gene expression profiles from our CN-AML patients. Using ours as a discovery set, we validated our results with three other independent CN-AML cohorts, which are available from public domains. Furthermore, we explored the possible molecular pathways underlying this signature.

## RESULTS

### Identification of genes with prognostic significance

**Figure 1:**
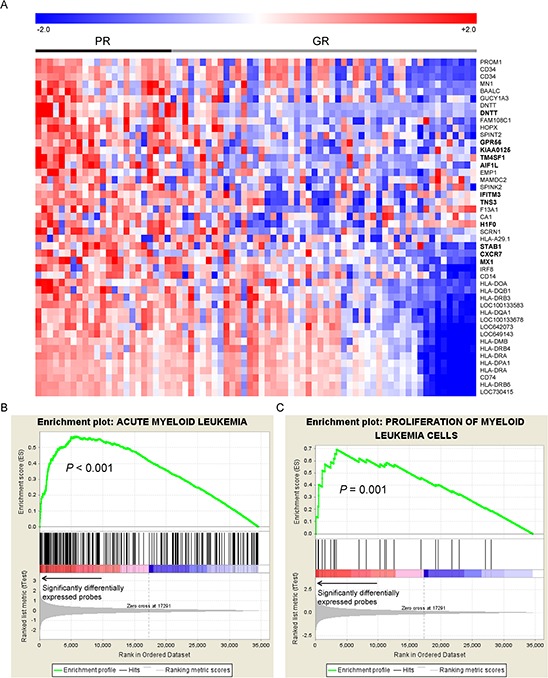
A. The heatmap of the 46 differential expressed probes between the 19 patients with poor response (PR group) to the first induction chemotherapy and the 56 achieving continuous complete remission (GR group) The 11 genes which were significantly associated with OS were highlighted in bold text. GSEA enrichment plots on genes associated with functions of **B.** acute myeloid leukemia and **C.** proliferation of myeloid cells are shown. The GSEA plots were used to confirm and visualize the significant terms reported by IPA. GSEA first ranked all genes probed on the microarray based on their significance in differential expression between PR and GR groups (denoted by an arrow in the figure). For a significant IPA term (component genes of which are denoted by black line segments), GSEA adopted a walking scoring method (green curve) to measure the degree to which the genes within an IPA term is overrepresented (i.e., enriched) to the left of all genes. Significance of the enrichment score was assessed by a permutation test. As a result, genes related to the two functions were significantly differentially expressed between the PR and GR groups, suggesting significant correlations between these two pathways and the treatment response.

We recruited a total of 351 adult patients (≥15 years of age) with newly diagnosed *de novo* AML from 1995 to 2011 at the National Taiwan University Hospital (NTUH), who had adequate cryopreserved bone marrow cells for mRNA array studies. Patients with antecedent hematological malignancies or therapy-related AML were excluded. We focused on the 158 patients (45.0%) with CN-AML. Among these patients, 104 (65.8%) received standard intensive chemotherapy. We analyzed the array data of the 158 CN-AML patients for global gene expression profiles. The expression data were processed and normalized to eliminate systematic biases and facilitate further statistical analyses. Since this study is a retrospective analysis with a group of patients spanning for almost 20 years, we aimed to eliminate biases as much as possible by using the response to induction chemotherapy as a criterion for dividing the patients into two groups, one with good response (GR group, 56 patients) who achieved continuous complete remission without relapse and the other with poor response (PR group, 19 patients) who were refractory to the induction chemotherapy. We compared gene expression profiles between the two groups and identified 46 differentially expressed probes (Student's *t*-test *P* value < 0.05 and > 2-fold change). These probes corresponded to 43 unique genes. Interestingly, all of the 46 probes were up-regulated in the PR group. Heatmap visualization of these probes were performed using the Genesis software (Fig. 1A) [23].

### Analysis of functional annotations of 43 genes

In order to dissect the biological functions underlying the 43 genes that likely affect chemosensitivity, we analyzed their functional annotations using the Ingenuity Pathway Analysis (IPA) [24] software. The genes were associated with abundant biological functions related to leukemia (data available upon request). Eight genes (*BAALC*, *CD14*, *CD34*, *CD74*, *DNTT*, *HLA*-*DRA*, *IRF8*, and *MN1*) were all associated with “leukemia” (*P* = 1.15 × 10^−4^), “acute myeloid leukemia” (*P* = 9.37 × 10^−3^), and “proliferation of myeloid cells” (*P* = 0.044). We further utilized Gene Set Enrichment Analysis (GSEA) [25] to verify the results derived from IPA. GSEA is an enrichment analysis algorithm that features threshold-free input (*i.e*., global gene profiles). It analyzes whether genes sharing a common function exhibit a global trend toward up-regulation (or down-regulation) in a given condition, measured by enrichment scores and permutation-based empirical *P*-values. Notably, genes related to the three associated terms (diseases and biological functions) obtained from IPA showed significant enrichment in the PR/GR differential gene expression profiles: the empirical *P*-values were <0.001 for functions related to acute myeloid leukemia and leukemia, and *P* = 0.001 for the proliferation of myeloid cells (enrichment plots in Fig. 1B–1C and Fig. S1). These 3 functional categories related genes contributed to a major fraction of the enrichment score, namely the leading-edge components. Appearing in leading-edge components of all three functions were ABL proto-oncogene 1 non-receptor tyrosine kinase (*ABL1*), B-cell CLL/lymphoma 2 (*BCL2*), and CD33 molecule (*CD33*).

### Construction of a risk scoring system

**Figure 2:**
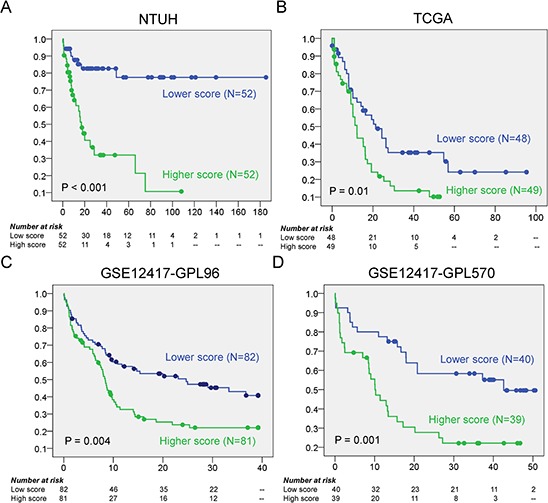
The Kaplan Meier curves for OS according to the scores **A.** In NTUH discovery set, patients with higher scores have significant shorter OS than those with lower scores (median 17.0 months vs. not reached, *P* < 0.001); **B–D.** In the three validation cohorts, the higher scores are all associated with poorer OS (median 12.2 vs 21.3 months, log rank *P* = 0.01 in TCGA; median 8.4 vs 24.7 months, log rank *P* = 0.004 in GSE12417-GPL96; median 10.1 vs 42.6 months, log rank *P* = 0.001 in GSE12417-GPL570).

In order to construct a risk scoring system, we analyzed the prognostic significance of expression of the 43 genes in survival. The survival analysis was conducted from the 104 patients (out of the 158 patients) who received standard intensive chemotherapy. Among the 46 probes associated with treatment response, 11 were significantly associated with OS (univariate Cox *P* < 0.01). These probes represented 11 unique genes (full gene list and results in Table 1; highlighted by boldface in Fig. 1A), and higher expression of each of these genes was associated with unfavorable prognosis (Kaplan Meier curves in Fig. S2). Based on the results we built a scoring system by incorporating the *z*-values (normalized gene expression levels, as defined in the Materials and Methods section) of the 11 genes with equal unity of weight to calculate a risk score for each patient. The risk score was significantly predictive of OS (univariate Cox *P* = 1.37 × 10^−6^ and log-rank *P* = 1.07 × 10^−5^; Fig. 2A), and disease free survival (DFS) (univariate Cox *P* = 1.16 × 10^−7^ and log-rank *P* = 9.71 × 10^−7^, Fig. S3). We further applied random permutation to evaluate the performance of our proposed scoring system against a random baseline (detailed in the Materials and Methods section). Remarkably, the scoring system outperformed all of ten-thousand random systems iteratively constructed by random selections of 11 genes from the dataset (empirical *P*-value <1.00 × 10^−4^, see the Materials and Methods section), suggesting the non-randomness of performance achieved by the proposed risk score.

**Table 1:** The list of 11 genes whose expression were significantly associated with overall survival among the 46 probes differential expressed between the patients with good and poor treatment response

Probe	Gene	Description	Univariate Cox *P*	Hazard ratio	95% confidence interval
1410021	*AIF1L*	allograft inflammatory factor 1-like	2.01E-03	1.657	1.203–2.284
450424	*CXCR7*	atypical chemokine receptor 3	1.00E-03	1.663	1.228–2.251
6280243	*DNTT*	DNA nucleotidylexotransferase	9.10E-03	1.471	1.101–1.966
5490768	*GPR56*	G protein-coupled receptor 56	6.86E-03	1.617	1.141–2.291
630278	*H1F0*	H1 histone family, member 0	7.03E-03	1.619	1.140–2.298
6650242	*IFITM3*	interferon induced transmembrane protein 3	1.96E-04	2.152	1.438–3.222
3780647	*KIAA0125*	KIAA0125	5.06E-03	1.558	1.143–2.125
1690066	*MX1*	MX dynamin-like GTPase 1	5.81E-03	1.618	1.150–2.279
6040053	*STAB1*	stabilin 1	7.39E-03	1.566	1.128–2.174
2680110	*TM4SF1*	transmembrane 4 L six family member 1	5.93E-05	1.869	1.377–2.537
5560561	*TNS3*	tensin 3	7.13E-04	2.133	1.376–3.308

For validation analysis we used three independent gene expression datasets from two studies, one from The Cancer Genome Atlas (TCGA) [26] and two (GSE12417-GPL96 and GSE12417-GPL570) from the study of Metzeler *et al*. [17]. The prognostic significance of higher risk score for unfavorable OS was validated by these independent cohorts of CN-AML (log rank *P* = 0.01, 0.004, and 0.001 in TCGA (*N* = 97), GSE12417-GPL96 (*N* = 163), and GSE12417-GPL570 (*N* = 79), respectively) (Fig. 2B, 2C and 2D).

We compared the performance of this 11-gene to the 7-gene risk scoring system proposed from another study [20]. Although the two scoring systems do not share any genes, they had equivalent prediction performance as shown by the similar *P* values in the three validation cohorts (Table 2). This was further confirmed by a multivariate analysis that incorporates these two scoring systems as co-variables (data not shown).

**Table 2:** Comparison between ours and the published 7-gene scoring system by univariate analysis

Dataset	11-gene risk score			7-gene unweighted score (Marcucci *et al*. 2014)		
	Hazard ratio	95% confidence interval	*P* value*	Hazard ratio	95% confidence interval	*P* value*
NTUH (*N* = 104)	1.12	1.07~1.18	1.4 × 10^−6^	1.29	1.06~1.56	0.012
TCGA (*N* = 97)	1.05	1.00~1.09	0.042	1.16	1.01~1.33	0.035
GSE12417-GPL96 (*N* = 163)	1.08	1.04~1.12	8.7 × 10^−5^	1.28	1.12~1.47	3.7 × 10^−4^
GSE12417-GPL570 (*N* = 79)	1.09	1.02~1.17	9.7 × 10^−3^	1.35	1.12~1.62	1.3 × 10^−3^

* Cox regression univariate analysis.

### Association of the scoring system with clinical and molecular characteristics

A higher risk score was positively associated with older age, lower count of white blood cells, and higher count of platelets (Table 3). FAB M1 leukemia occurred less frequently in the higher score group. The profiles of genetic mutations were significantly different between higher and lower score groups: patients with higher scores more often had *FLT3*-ITD, *RUNX1*, *MLL*-PTD, *ASXL1*, and *DNMT3A* mutations, but less likely had *NPM1* and *CEBPA* mutations (Table 4). In particular, nearly all *CEBPA*-mutated patients were in the lower score group, whereas all *MLL*-PTD mutated patients were in the higher score group.

**Table 3:** Correlation between mRNA score and clinical and laboratory features in CN-AML patients (*n* = 158)

Variant	Total	mRNA Score		*P*
		*Low (n = 79)*	*High (n = 79)*	
Age* (years)	58 (16–90)	55 (18–87)	62 (16–90)	0.027
Age, in groups				
>60	76 (48.1%)	32 (40.5%)	44 (55.7%)	0.056
>50	97 (61.4%)	43 (54.4%)	54 (68.4%)	0.072
Gender				
male	90 (57.0%)	44 (55.7%)	46 (58.2%)	0.748
Lab data*				
WBC (×10^3^/μL)	28.88 (0.65–423.0)	41.38 (0.98–423.0)	24.72 (0.65–341.4)	0.042
Blasts (×10^3^/μL)	13.77 (0–342.1)	18.69 (0–342.1)	10.78 (0–310.7)	0.056
Hemoglobin, g/dL	8.1 (3.7–14.0)	8.3 (4.2–14.0)	7.9 (3.7–13.2)	0.173
Platelets (×10^3^/μL)	53.0 (6–331)	42.0 (6–214)	60.0 (9–331)	0.006
LDH (U/L)	878.0 (274–13130)	960.0 (354–13130)	804.0 (274–7177)	0.053
FAB				0.156
M0	2 (1.3%)	0	2 (2.5%)	0.155
M1	37 (23.4%)	24 (30.4%)	13 (16.5%)	0.039
M2	53 (33.5%)	28 (35.4%)	25 (31.6%)	0.613
M4	52 (32.9%)	22 (27.8%)	30 (38.0%)	0.176
M5	12 (7.6%)	4 (5.1%)	8 (10.1%)	0.230
M6	2 (1.3%)	1 (1.3%)	1 (1.3%)	>0.999

* median (range)

**Table 4:** Correlation of mRNA score with other gene alterations

Mutation	Total (*n* = 158)	*mRNA Score*		*P*
		*Lower (n = 79)*	*Higher (n = 79)*	
*NPM1*	78 (49.4%)	47 (59.5%)	31 (39.2%)	0.011
*FLT3-*ITD	52 (32.9%)	20 (25.3%)	32 (40.5%)	0.042
*NPM1*^+^/*FLT3-*ITD^−^	45 (28.5%)	32 (40.5%)	13 (16.5%)	0.001
*CEBPA*^double^	21 (13.5%)	20 (25.6%)	1 (1.3%)	<0.001
*CEBPA*	30 (19.2%)	29 (37.2%)	1 (1.3%)	<0.001
*WT1*	13 (8.3%)	4 (5.1%)	9 (11.5%)	0.148
*RUNX1*	26 (16.6%)	2 (2.6%)	24 (30.4%)	<0.001
*IDH1*	12 (7.7%)	6 (7.7%)	6 (7.7%)	>0.999
*IDH2*	28 (17.9%)	18 (23.1%)	10 (12.8%)	0.095
*FLT3-*TKD	14 (9.0%)	7 (9.0%)	7 (9.0%)	>0.999
*MLL-*PTD	10 (6.5%)	0	10 (12.8%)	0.001
*KIT*	2 (1.3%)	2 (2.6%)	0	0.155
*KRAS*	3 (1.9%)	1 (1.3%)	2 (2.6%)	0.560
*NRAS*	24 (15.4%)	14 (17.9%)	10 (12.8%)	0.375
*ASXL1*	20 (12.7%)	3 (3.8%)	17 (21.5%)	0.001
*TET2*	31 (19.9%)	15 (19.2%)	16 (20.5%)	0.841
*DNMT3A*	40 (25.6%)	13 (16.7%)	27 (34.6%)	0.010

### Survival analysis

The univariate analysis of the clinical parameters and molecular alterations on OS in our CN-AML patients was shown in Table S1. Since higher risk scores seemed to be highly associated with other poor prognostic variables, we sought to investigate whether our scoring system functioned as an independent factor. We included variables that were significantly associated with clinical outcome from univariate analysis, including age, ELN (European LeukemiaNet) genetic group, *MLL*, *RUNX1*, *TET2* mutations, and mRNA score, for multivariate analysis. We found higher scores appeared to be a strong independent risk factor (Table 5).

**Table 5:** Multivariate analysis (Cox regression) for the OS in CN-AML cohort

Variables	Hazard ratio	95% confidence interval	*P* value
Age	1.030	1.006~1.054	0.012
ELN genetic group¶	1.191	0.472~3.004	0.711
*MLL*^#^	1.058	0.289~3.868	0.932
*RUNX1*^#^	0.653	0.261~1.635	0.363
*TET2*^#^	2.876	1.234~6.701	0.014
mRNA score	1.146	1.061~1.238	0.001

¶ ELN favorable risk vs. Intermediate-1 risk

# mutated vs wild

### Biological functions associated with the scoring system

**Figure 3:**
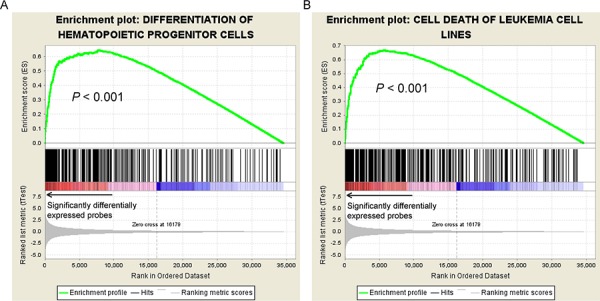
GSEA enrichment plots on genes associated with **A.** differentiation of hematopoietic progenitor cells and **B.** cell death of leukemic cell lines. Genes related to these two functions were significantly differentially expressed between the patients with higher and lower mRNA scores, suggesting significant correlations between these two pathways and the scoring.

To gain biological insights into the risk scores, we further analyzed genes that were differentially expressed in patients with higher or lower risk scores. Patients with risk scores above and below the average by one standard deviation in the NTUH dataset were defined as the high-risk and low-risk groups, respectively. We identified 578 differentially expressed probes (Student's *t*-test *P* < 0.05 and >2-fold change) that corresponded to 509 unique genes. In the list, we identified some homeobox genes up-regulated in high-risk patients, including *HOXA3* (*t*-test *P* = 4.56 × 10^−5^), *HOXA5* (*P* = 2.73 × 10^−7^), *HOXA6* (*P* = 3.34 × 10^−7^), *HOXA9* (*P* = 4.72 × 10^−8^), *HOXA10* (*P* = 5.83 × 10^−8^), *HOXB2* (*P* = 5.95 × 10^−8^), *HOXB3* (*P* = 5.61 × 10^−5^), *HOXB4* (*P* = 1.37 × 10^−6^), *HOXB5* (*P* = 3.90 × 10^−6^), *HOXB6* (*P* = 2.65 × 10^−3^), *HOXB7* (*P* = 2.21 × 10^−3^), *HOXB8* (*P* = 1.78 × 10^−2^), *MEIS1* (*P* = 6.18 × 10^−7^), and *PBX3* (*P* = 5.02 × 10^−4^). The homeobox genes are well known for their crucial functions in stemness maintenance, and adverse prognosis in AML when their expression levels are elevated [15, 27, 28]. Furthermore, IPA revealed significant associations between the 509 differentially expressed genes with abundant important biological functions in AML (data available upon request), including proliferation of blood cells (*P* = 2.25 × 10^−10^), cell death of leukemia cell lines (*P* = 2.41 × 10^−9^), differentiation of hematopoietic progenitor cells (*P* = 9.85 × 10^−7^), and quantity of hematopoietic progenitor cells (*P* = 4.06 × 10^−5^). All of these functions were validated with significant enrichment by GSEA (empirical *P*-values all < 0.001; enrichment plots in Fig. 3 and Fig. S4). Taken together, our data indicate that the 11-gene scoring system modulates the treatment response of CN-AML patients through regulation of several crucial cellular functions.

## DISCUSSION

In this study, we grouped the patients by their responses to induction chemotherapy in order to identify genes related to drug sensitivity. By this approach, we aimed to eliminate potential biases raised from our retrospective cohort. IPA analysis showed that 43 differentially expressed genes were closely related to biological functions associated with “leukemia”, “acute myeloid leukemia”, and “proliferation of myeloid cells”. Moreover, using GSEA, we found *ABL1, BCL2, and CD33* were among leading-edge components of all three functional categories. Expression of *BCL2*, both at transcriptional and translational levels, is known to correlate with poor treatment responses to chemotherapy and low complete remission rates in AML [29–31]. While *ABL1* has been relatively unexplored in AML, it has been implied for association with the resistance of chemotherapy in chronic myeloid leukemia [32]. Our data indicated these genes may be highly involved in crucial biological functions that determined treatment response.

A high risk score was associated with unfavorable mutations (*FLT3*-ITD, *MLL*-PTD, *ASXL1*, *RUNX1*, and *DNMT3A* mutations) but inversely associated with favorable ones (*NPM1* and *CEBPA* mutations). Radmacher *et al*. showed a strong correlation between the prognostic classifier and the status of *FLT3*-ITD, but not that of *MLL*-PTD, *NPM1*, or *CEBPA* mutations [16]. In addition, Metzeler *et al*. demonstrated that a high risk signature was associated with *FLT3*-ITD, *WT1*, *RUNX1*, and *TET2* mutations, but inversely associated with *CEBPA* mutations [18]. Nevertheless, we demonstrated the independence of our signature from other important prognostic factors. These observations suggest that our scoring system bears similar biological implications to other parameters on clinical outcome yet stands alone as a new tool for prediction of treatment response.

We constructed a simple mRNA signature as a prognostication tool based on the expression levels of 11 genes for CN-AML patients, a subgroup in which the requirement of prognostic parameters is yet unmet. Several study groups have developed gene expression signature for predicting prognosis in AML patients. In six studies [15–20, 33], the results were validated by independent datasets, as in ours. The 11 gene signature of our mRNA scoring system included *GPR56, KIAA0125, TM4SF1, AIF1L*, *CXCR7*, *DNTT*, *H1F0*, *IFITM3*, *MX1*, *STAB1*, and *TNS3*; only the first three were seen in another mRNA signature [17], reflecting the variations in study populations or treatment protocols. Although there was no overlap of the genes between our proposed scoring system and the 7-gene signature proposed from an epigenetic study [20] or the 24-gene score improving the ELN classification [33], our system-derived scores were moderately correlated with the scores from the two studies (correlation coefficients, 0.68 and 0.45, respectively, data not shown). Such concordance implies these prognostic scores, though derived from different analysis schemes, might represent common underlying biological mechanisms. Future study may further address this in a larger AML cohort. Current knowledge about the association between the 11 genes and malignancies is summarized in Table 6. In some studies aberrant expression of *CXCR7* [34–36], *DNTT* [37, 38], *GPR56* [39], *H1F0* [40], and *MX1* [41] is seen in leukemia. Little is known about the role of *AIF1L* and *KIAA0125* [42] in pathogenesis of cancers. *IFITM3* [43], *MX1* [44–46], *STAB1* [47], *TM4SF1* [48, 49], and *TNS3* [50–52] may have roles in various solid cancers, but they are not yet explored in AML. *STAB1* [47], *TM4SF1* [49], and *TNS3* [52] are involved in cell adhesion and motility, which are relevant to cancer metastasis and invasion, however, their roles in interaction between leukemic stem cells and bone marrow niche deserve further investigation.

**Table 6:** Summary of the association between 11 genes and malignancy

Probe	Gene	Association with leukemia or solid cancers
**1410021**	*AIF1L*	No data
**450424**	*CXCR7*	Essential for the survival and growth of tumor cells [34]; Highly expressed in several human myeloid malignant cell lines [35]; Overexpressed in CN-AML patients with adverse clinical outcomes [36]
**6280243**	*DNTT*	Lymphoid regulator, up-regulated in *RUNX1*-mutated CN-AML [37, 38]
**5490768**	GPR56*	Influencing adhesion, migration, homing and mobilization of AML stem cells through the RhoA signaling pathway, especially in *EVI1* over-expressed leukemia [39]
**630278**	*H1F0*	Important for murine erythroleukemia cell differentiation [40]
**6650242**	*IFITM3*	Overexpression in gastric cancer [43]
**3780647**	KIAA0125*	Not reported yet in cancers, but involved in neurogenesis and the pathogenesis of Alzheimer's disease [42]
**1690066**	*MX1*	Diminished expression in AML [41]; Up-regulated in lymph node-positive colorectal cancer [44]; Down-regulated in renal cell carcinoma [45] and head and neck cancers [46]
**6040053**	*STAB1*	Cell adhesion and motility [47]
**2680110**	TM4SF1*	Prostate cancer [48]; Pancreatic cancer, cell adhesion and motility [49]
**5560561**	*TNS3*	Renal cell carcinoma [50, 51]; Breast cancer, cell adhesion and motility [52]

* genes also seen in the classifier of Metzeler *et al*. [17]

In conclusion, we present a simple mRNA expression scoring system for prognostication of CN-AML. The scoring system was validated by three independent cohorts and has comparable performance as the system proposed by Marcucci *et al.* [20]. Our scoring system is composed of only 11 genes, making it highly potential in clinical use. Its positive association with multiple clinically relevant gene mutations suggests that it has incorporated the prognostic implications of multiple conventional risk factors. Our scoring system may provide another prognostic reference other than genetic mutations currently used for CN-AML. However, a large prospective cohort in which a q-PCR-based measurement of the expression of the 11 genes is necessary for clinical application of this scoring system.

## MATERIALS AND METHODS

### Patients

We recruited a total of 351 adult patients (≥15 years of age) with newly diagnosed *de novo* AML from 1995 to 2011 at the National Taiwan University Hospital (NTUH), who had adequate cryopreserved bone marrow cells for mRNA array studies. Patients with antecedent hematological malignancies or therapy-related AML were excluded. The bone marrow specimens were harvested by bone marrow aspiration. The mononuclear cells were isolated by treating with Ficoll-Paque. This study was performed in accordance with the Declaration of Helsinki and was approved by the Research Ethics Committee of the NTUH. We focused on the 158 patients (45.0%) with CN-AML. Among these patients, 104 (65.8%) received standard intensive chemotherapy as described previously [53]. Briefly, they received induction chemotherapy (idarubicin 12 mg/m^2^ per day on days 1 to 3 and cytarabine 100 mg/m^2^ per day on days 1 to 7) and then consolidation chemotherapy with 2 to 4 courses of high-dose cytarabine (2000 mg/m^2^ every 12 hours; total 8 doses), with or without an anthracycline (idarubicin or mitoxantrone), after achieving complete remission (CR). The remaining 54 patients were treated with palliative care or low-dose chemotherapy due to patients' preference or poor performance status. All 158 patients were included for analyses of correlation between the risk score and clinical and other biological parameters, but only the 104 patients who received standard intensive chemotherapy were included for survival analysis. Forty of the 104 patients received allogeneic hematopoietic stem cell transplantation (HSCT); they were censored on the day of stem cell infusion to avoid confounding factors brought by the HSCT therapy. In the survival analysis, >90% statistical power can be achieved based on a sample of at least 93 patients (at a 0.01 significance level to detect a hazard ratio of 2; calculated by PASS software (NCSS, Kaysville, Utah)).

### Cytogenetic and mutation analysis

Chromosomal abnormalities and gene mutations were analyzed as described previously [3, 53–56].

### mRNA microarray analysis and data processing

We profiled whole-genome gene expression of 158 patients using Illumina HumanHT-12 v4 Expression BeadChip (Illumina, San Diego, CA), following the manufacturer's instructions. Briefly, we verified RNA concentration and integrity with ND-1000 spectrophotometer (NanoDrop Technologies, Wilmington, DE) and 2100 Bioanalyzer (Agilent Technologies, Palo Alto, CA). 1.5 μg cRNA of each sample was hybridized to Illumina HumanHT-12 v4 Expression BeadChip. Intensities of bead fluorescence were detected with Illumina BeadArray Reader (Illumina, San Diego, CA), followed by transformation to numeric values using GenomeStudio v2010.1 software (Illumina, San Diego, CA). We performed Log-2 transformation and quantile normalization for the data to achieve normalized probe-level expression values. The microarray data have been deposited in the Gene Expression Omnibus (accession number: GSE71014).

### External dataset processing

For validation analysis we included three independent gene expression datasets from two studies, one from The Cancer Genome Atlas (TCGA) [26] and two (GSE12417-GPL96 and GSE12417-GPL570) from the study of Metzeler *et al*. [17]. The TCGA dataset was composed of gene expression profiles from 197 AML samples (97 CN) achieved by Affymetrix Human Genome U133 Plus 2.0 Array. Level-2 data, which were probe-level pre-normalized signals processed by TCGA, were downloaded and transformed into Log-2 scale. The two datasets from the study by Metzeler *et al*., including 163 and 79 CN-AML patients, respectively, were profiled with Affymetrix Human Genome U133 Plus 2.0 Array. We used the authors' pre-processed datasets deposited in the Gene Expression Omnibus (GEO, accession ID GSE12417) [57]. In the three datasets, each of the genes with multiple probes was represented by the most “informative” probe that carried the largest coefficient of variation, defined as the ratio of per-probe standard deviation to per-probe average.

### Statistical analysis

Statistical significance of differential expression of genes between two groups of samples was assessed using Student's *t*-test. For survival analysis, expression values of each dataset were first *z*-transformed (*i.e*., subtraction of sample mean followed by division by sample standard deviation for each probe) to approximately follow the normal distribution (zero mean and unity standard deviation). We then utilized the univariate Cox proportional hazards model to determine association between expression of individual genes and patient survival.

We employed a ten-thousand-time random permutation test to evaluate the performance and randomness of constructed risk scoring system with the process as described previously [58]. Briefly, in each of the ten thousand iterations a random system was constructed by substituting the genes in the proposed scoring system with randomly selected ones from the microarray dataset. Each random system was tested for survival significance. After all iterations, significance of the proposed system was measured by the empirical *P*-value, which was simply the fraction of random risk systems that achieved higher Cox significance than the proposed system.

We adopted Kaplan-Meier estimation to plot survival curves and used log-rank tests to examine the difference between groups. The patients who received allogeneic HSCT were censored on the day of cell infusion. Hazard ratio and 95% confidence interval were estimated by Cox proportional hazards regression models to determine independent risk factors associated with survival in multivariate analyses. For analysis of differential expression, two-sided *P* values from Student's *t*-test less than 0.05 were considered statistically significant. The whole patient population was included for analyses of correlation between the risk score and clinical characteristics and molecular alternations; however, only those receiving conventional standard chemotherapy, as mentioned above, were included in analyses of survivals.

### Functional annotation analysis

In order to gain biological insights into identified groups of genes, we further incorporated two functional annotation tools, Ingenuity Pathway Analysis (IPA; Qiagen, Redwood City, CA) [24] and Gene Set Enrichment Analysis (GSEA, Java program downloadable athttp://www.broadinstitute.org/gsea/index.jsp) [25]. IPA is a knowledge-based database that features manual curation of a huge volume of published literatures. It employs Fisher's exact test to assess the significance of association between biological functions and the set of genes of interest (*e.g*. differentially expressed genes). We used GSEA to further verify the results of IPA. Instead of analyzing sets of genes of interest, GSEA is designed to detect whether a biological function is enriched in the whole-genome expression pattern; *i.e*., significant *P*-value from GSEA indicates significant overall enrichment of genes sharing a common function in genes with differential expressions. Here the gene sets (biological functions) were downloaded from the IPA database. Significance of enrichment was assessed based on the two-thousand-time random permutation test among genes.

## SUPPLEMENTARY FIGURES AND TABLES


