# Supplementary material for: An mRNA expression signature for prognostication in de novo acute myeloid leukemia patients with normal karyotype
Source: Oncotarget. 2015 Oct 23;6(36):39098–110. doi: 10.18632/oncotarget.5390 (PMC4770759; doi:10.18632/oncotarget.5390)
Supplement: Supplementary file 1 [file oncotarget-06-39098-s001.pdf]

SUPPLEMENTARY FIGURES AND TABLE

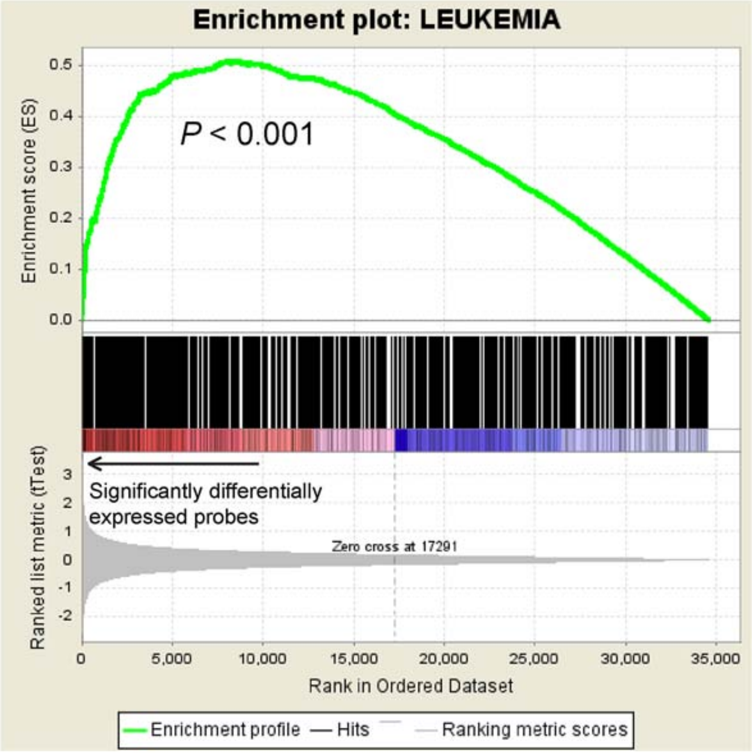

Supplementary Figure S1: GSEA enrichment plots on genes associated with leukemia. Genes related to this function were significantly differentially expressed between the PR and GR groups, suggesting significant correlations between this pathway and the treatment response.

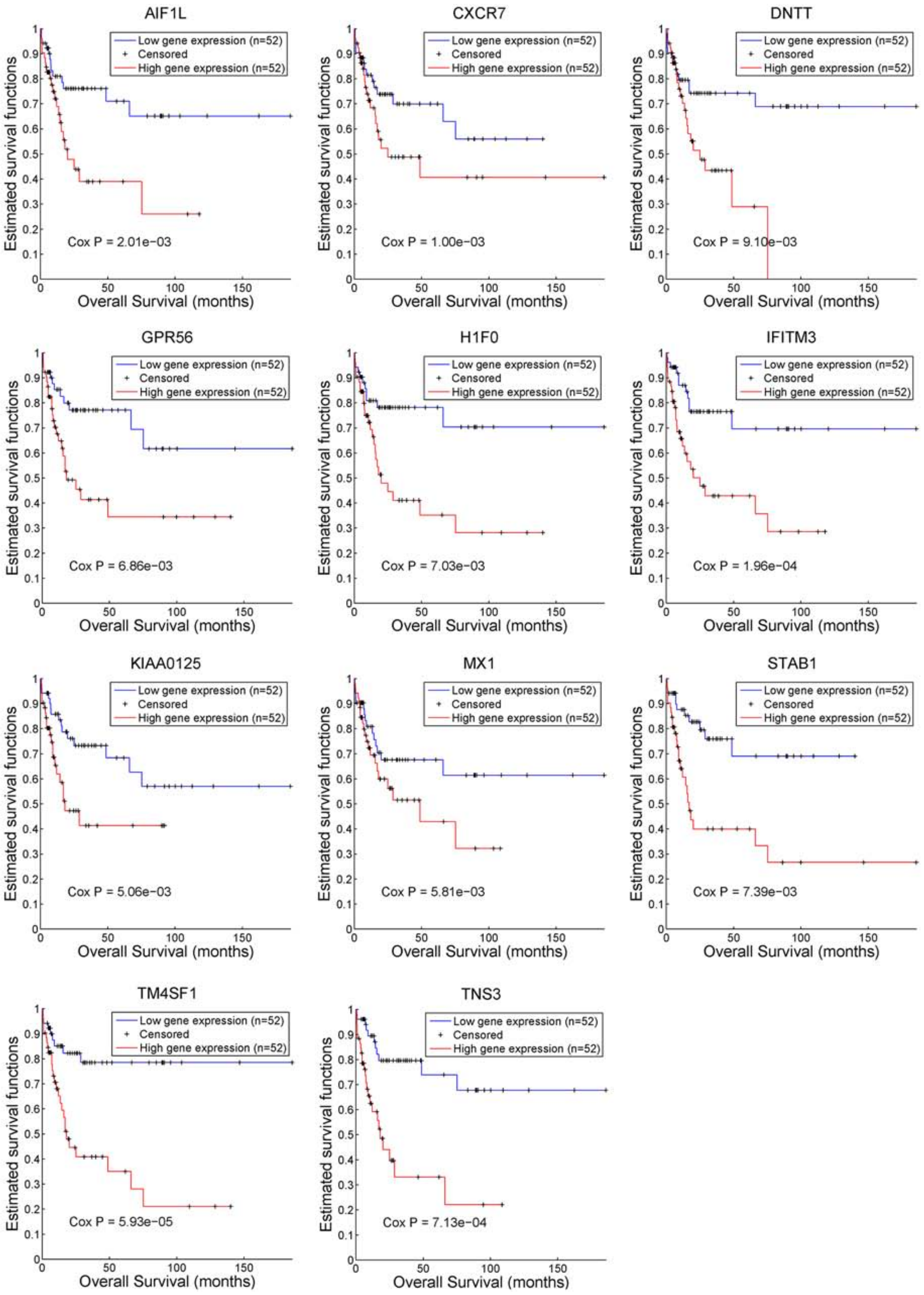

Supplementary Figure S2: The Kaplan Meier curves for OS stratified according to the expression levels of 11 genes which were significantly associated with OS among the 46 probes differentially expressed between the patients with good and poor response.

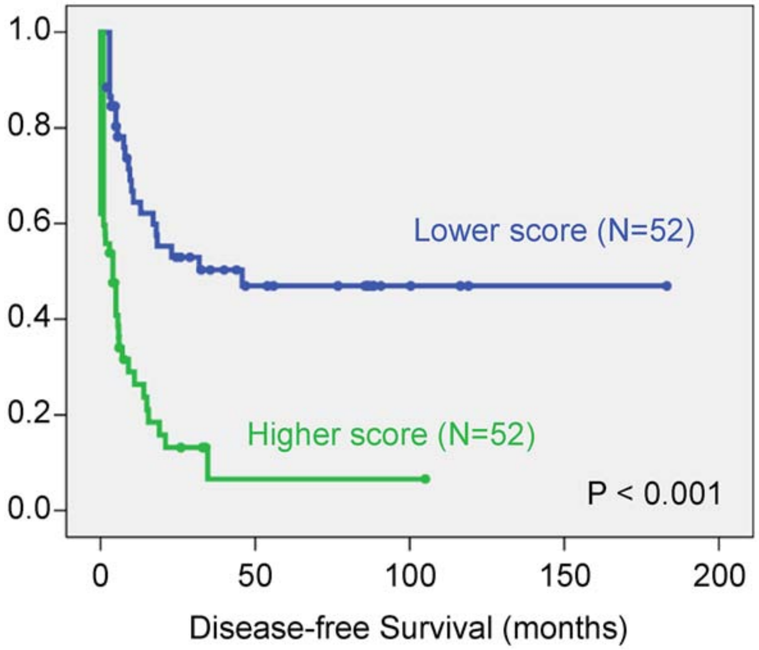

Supplementary Figure S3: The Kaplan Meier curves for DFS according to the scores in NTUH cohort. The patients with higher scores have significant shorter DFS than those with lower scores (median 4.0 vs. 45.8 months, log-rank $P = 9.71 \times 10^{-7}$)

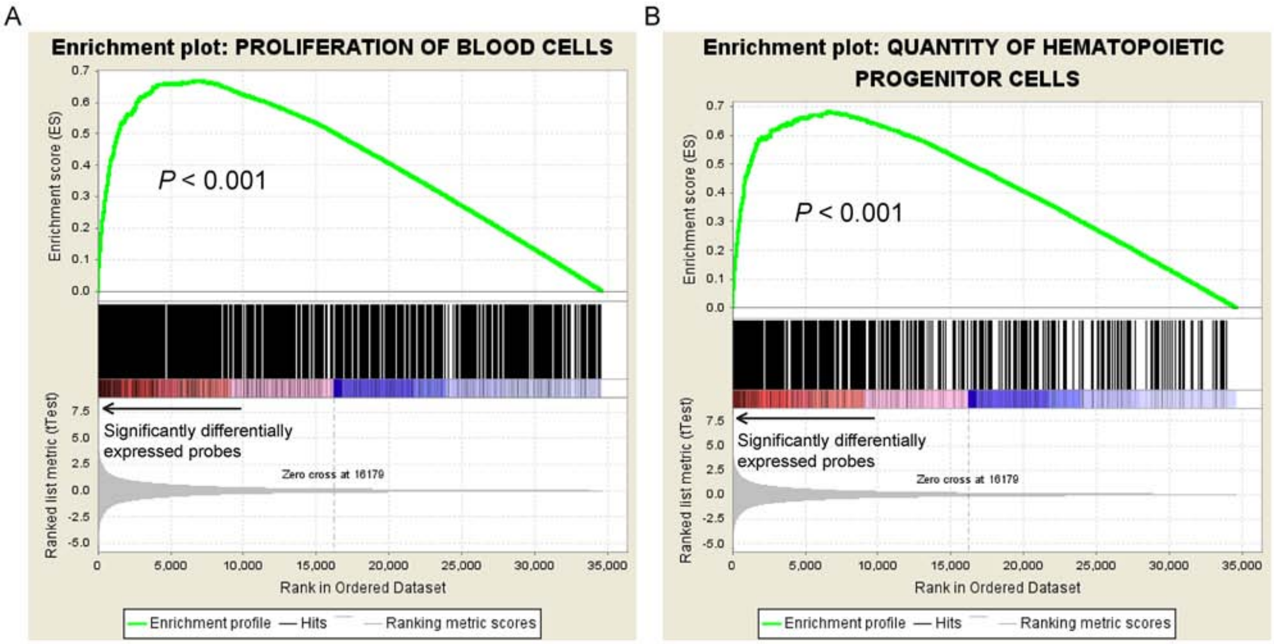

Supplementary Figure S4: GSEA enrichment plots on genes associated with A. proliferation of blood cells and **B.** quantity of hematopoietic progenitor cells. Genes related to these two functions were significantly differentially expressed between the patients with higher and lower mRNA scores, suggesting significant correlations between these two pathways and the scoring.

Supplementary Table S1: Univariate analysis of the impact of clinical parameters and molecular alterations on overall survival in CN-AML patients

variable	Overall Survival	
	Median (months)	*P* value
Age		0.005
≤50	NR	
>50	28.7	
WBC		0.529
≤50 k/μL	75.2	
>50 k/μL	NR	
CEBPA		0.010
Double	NR	
Others	66.1	
NPM1		0.334
Mutated	NR	
Wild	66.1	
FLT3-ITD		0.007
Mutated	15.5	
Wild	NR	
NPM1+/*FLT3*-ITD-		0.582
Compatible	NR	
Others	NR	
ELN Genetic Group		0.001
Favorable	NR	
Intermediate-1	18.0	
MLL-PTD		0.023
Mutated	10.5	
Wild	66.1	
RUNX1		0.012
Mutated	18.0	
Wild	NR	
WT1		0.237
Mutated	14.5	
Wild	NR	
IDH1		0.728
Mutated	NR	
Wild	NR	

(*Continued*)

variable	Overall Survival	
	**Median (months)**	***P* value**
IDH2		0.270
Mutated	75.2	
Wild	NR	
FLT3-TKD		0.345
Mutated	25.0	
Wild	NR	
N-RAS		0.765
Mutated	66.1	
Wild	NR	
ASXL1		0.583
Mutated	16.0	
Wild	NR	
TET2		0.011
Mutated	9.0	
Wild	NR	
DNMT3A		0.223
Mutated	75.2	
Wild	NR	
mRNA score		<0.001
Lower	NR	
Higher	17.0	

Abbreviation: NR, not reached
